# Atypical Presentation of Pancreatic Cancer Mimicking IgG4-Related Disease

**DOI:** 10.7759/cureus.85876

**Published:** 2025-06-12

**Authors:** Yazan Sallam, Mir Zulqarnain, Anas Al-sadi, Chase Branstetter, Asher Mir

**Affiliations:** 1 Internal Medicine, University of Missouri Kansas City, Kansas City, USA; 2 Gastroenterology, University of Missouri Kansas City, Kansas City, USA; 3 Internal Medicine, Chicago Medical School, North Chicago, USA

**Keywords:** autoimmune pancreatitis mimicker, malignant pancreatic cancer, massless pancreatic cancer, pancreatic metastasis, supraclavicular lymph node

## Abstract

Pancreatic cancer often presents with vague, nonspecific symptoms such as painless jaundice, weight loss, and fatigue. Imaging can be diagnostic in pancreatic cancer. However, in rare cases, pancreatic cancer may present without a detectable mass, mimicking conditions like autoimmune pancreatitis (AIP). Supraclavicular lymph node metastasis, commonly associated with breast, lung, gastric, and esophageal cancers, is rarely reported in cases of pancreatic cancer. Even more unusual is the finding of an isolated Virchow’s node in such cases. Here, we present a unique case of pancreatic adenocarcinoma characterized by an isolated, enlarged left supraclavicular lymph node without any clear evidence of a pancreatic mass on imaging.

## Introduction

The presentation of pancreatic cancer is often vague, with nonspecific symptoms such as painless jaundice, weight loss, and fatigue [1]. The diagnosis is commonly made after a pancreatic mass is identified via radiography. Rarely, this malignancy can present without a discrete mass and can mimic autoimmune pancreatitis (AIP). Additionally, although supraclavicular lymph node metastasis is typically seen with breast, lung, gastric, and esophageal malignancies, the findings of an isolated Virchow’s node (spread through the lymphatic system) are rarely described in the literature [2]. Here we describe an unusual case of pancreatic adenocarcinoma with radiographic evidence of an isolated, enlarged left supraclavicular lymph node without obvious pancreatic mass.

## Case presentation

**Figure 1:**
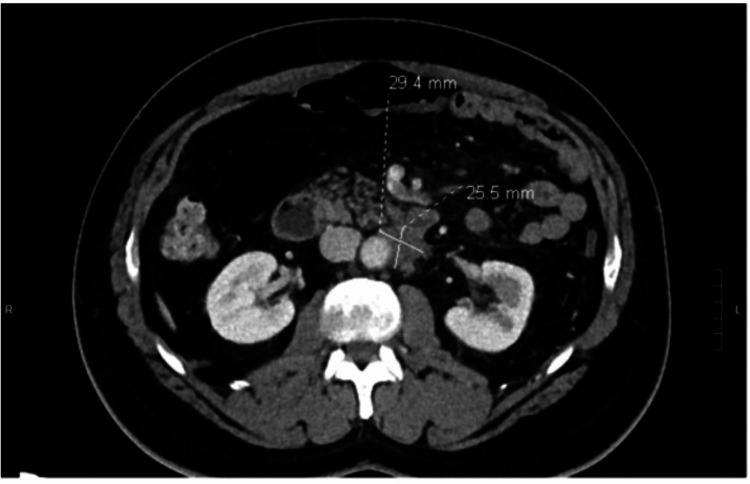
Hypo-enhancement and loss of fatty clefts at the distal body and tail of the pancreas without obvious mass.

**Figure 2:**
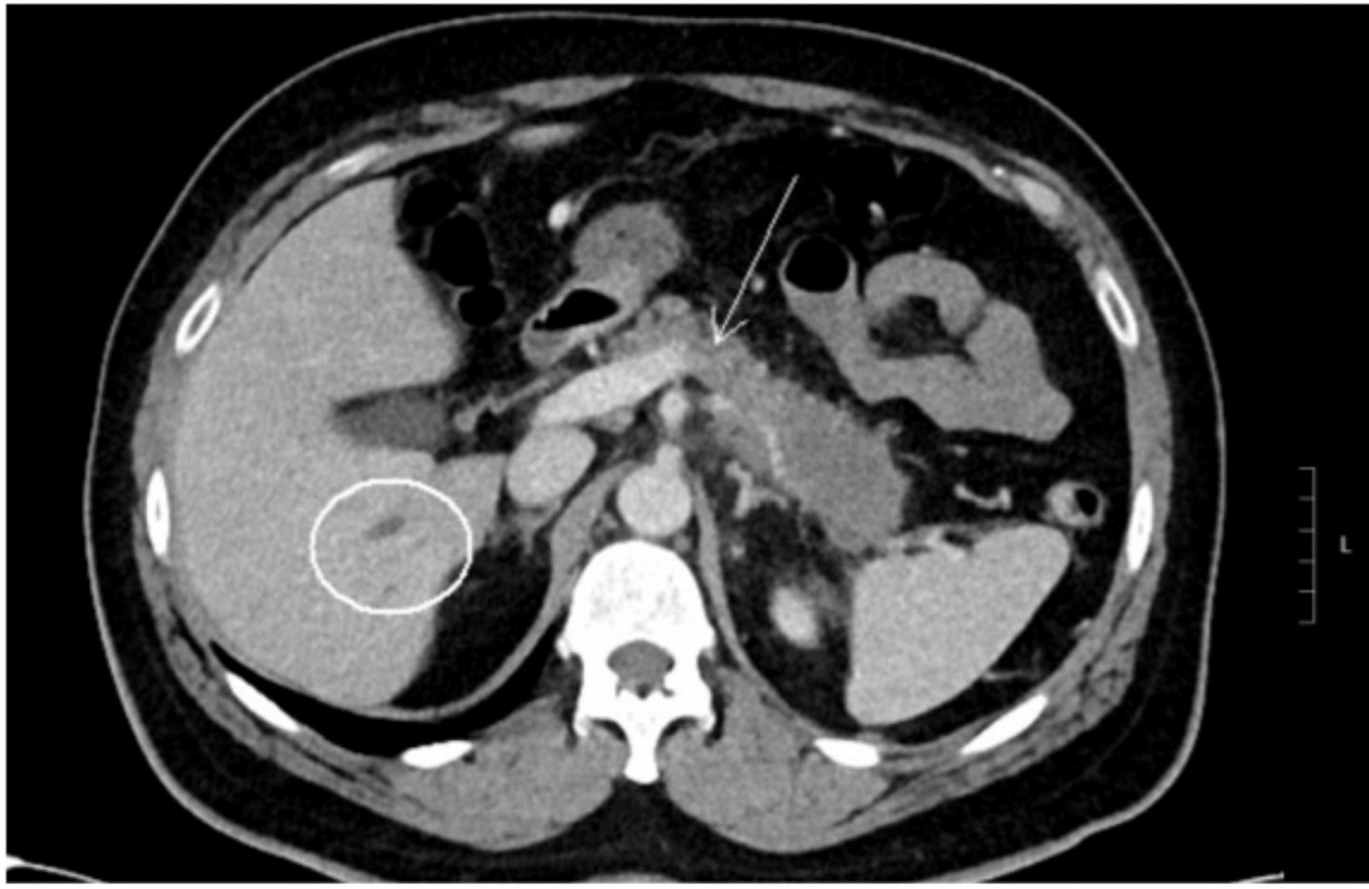
Arrow pointing to nodularity of the retroperitoneal soft tissue concerning for retroperitoneal fibrosis.

**Figure 3:**
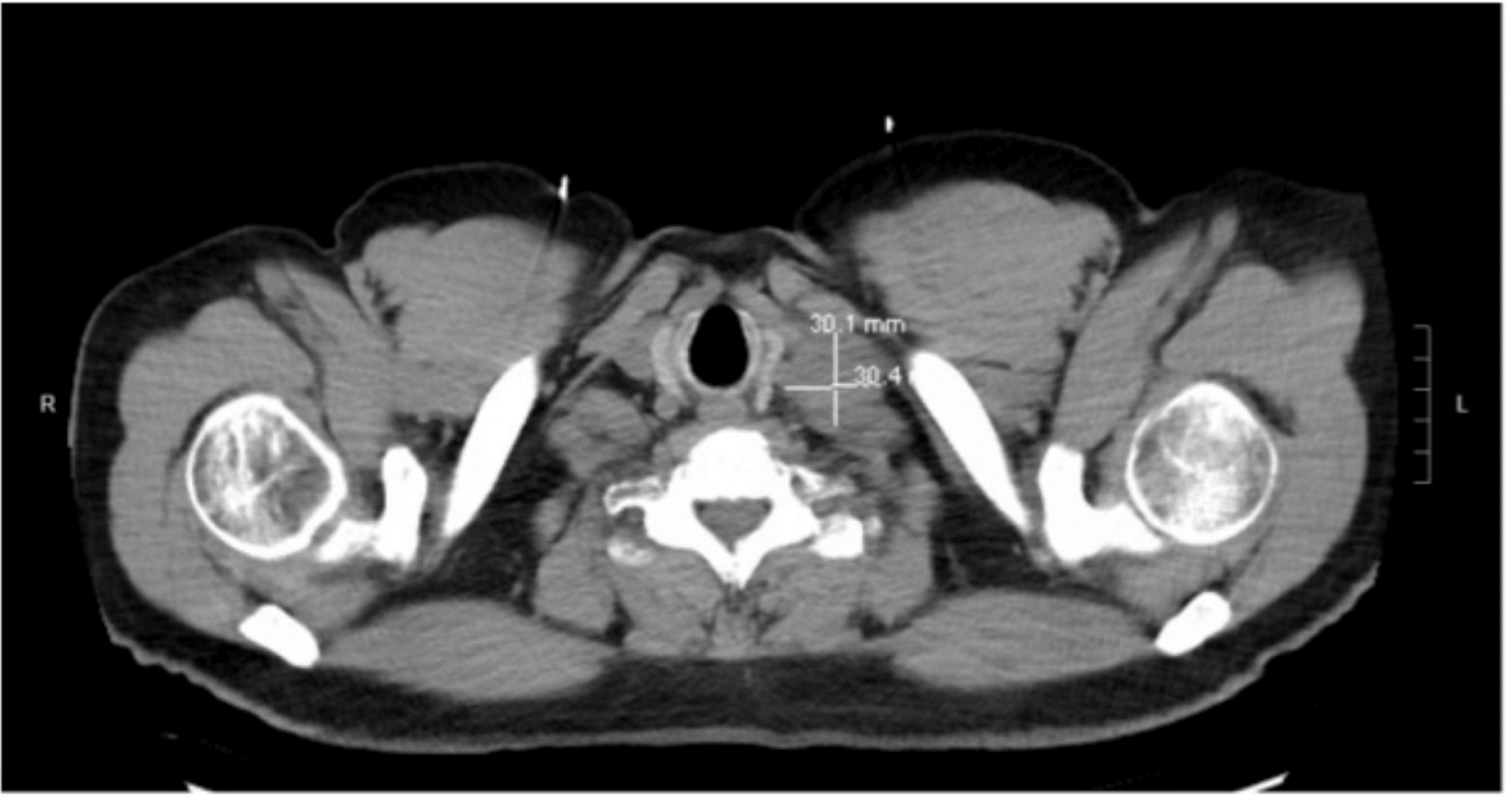
Ill-defined left supraclavicular mass.

A 61-year-old man presented to a tertiary medical center with dull, constant abdominal pain radiating to his back over the last month. He was vitally stable, and labs were unremarkable, with normal complete blood count (CBC), liver function tests (LFT), and lipase. Detailed lab values are in Table 1. He underwent a computed tomography (CT) scan of the abdomen while in the emergency room which showed hypo-enhancement and loss of fatty clefts at the distal body and tail of the pancreas without obvious mass (Figure 1), with the CT findings, along with his abdominal pain, initial impression of pancreatitis was made. Other findings on the CT included occlusion of the splenic vein and nodularity of the retroperitoneal soft tissue, concerning for retroperitoneal fibrosis (Figure 2). The workup to this point strongly suggested an autoimmune process, such as IgG4-related AIP, given the constellation of symptoms and findings on imaging. However, IgG4 levels were checked and were only modestly elevated at 102 mg/dL (Reference 2 mg/dL - 96 mg/dL). The patient underwent endoscopic ultrasound (EUS) with core-needle, pancreatic biopsies in hopes of detecting autoimmune changes and IgG4 staining; instead, the biopsy revealed invasive moderately differentiated adenocarcinoma. A chest CT was obtained for staging, and it revealed an ill-defined left supraclavicular mass that was concerning for metastasis to a lymph node (Figure 3). It is worth mentioning that this lymph node was not detectable on clinical examination. Interventional radiology (IR) performed a biopsy of the lymph node, which resulted in a metastatic moderately differentiated adenocarcinoma that was consistent with pancreatic origin. The patient was seen by the inpatient oncology service with plans to initiate palliative chemotherapy for stage IV disease with FOLFIRINOX (Folinic acid, Fluorouracil, Irinotecan, Oxaliplatin).

**Table 1:** Lab values ALT: alanine transaminase; AST: aspartate transaminase; ALP: alkaline phosphatase

Test	Value	Reference Range
Hb	14 g/dL	13 - 17 g/dL
WBC	8 TH/uL	4 - 11 TH/uL
Platelet count	290 TH/uL	140 - 400 TH/uL
Albumin	4 g/dL	3.3 - 5 g/dL
ALT	15 U/L	0 - 49 U/L
AST	10 U/L	0 - 34 U/L
ALP	55 U/L	44 - 150 U/L
Total bilirubin	0.3 mg/dL	0.2 - 1.1 mg/dL
Lipase	25 U/L	12 - 53 U/L
IgG4	102 mg/dL	2 - 96 mg/dL
CA 19-9	96 U/mL	<37 U/mL

## Discussion

Pancreatic cancer has the highest mortality rate among all cancers, as well as being the third leading cause of all cancer-related mortality in the United States (US), with lung and colon occupying the first and second places, respectively [3]. Most patients with pancreatic cancer are diagnosed at an advanced stage, which contributes to the high mortality rate for this cancer [4]. The overall five-year survival is estimated to be around 6% [5]. Risk factors for pancreatic cancer include hereditary factors, which can increase the risk up to 132-fold compared to the normal population, along with environmental risk factors. Some of the environmental risk factors include smoking, alcohol, obesity, diabetes, diet, and chronic pancreatitis [6].

Pancreatic cancer is usually diagnosed in its later stages, often when distant metastasis has already occurred. The most common sites for metastasis include the liver. Other common sites include the peritoneum, stomach, and testes [7]. While still under investigation, the different metastatic patterns of pancreatic cancer are speculated to affect the outcome and prognosis of the disease [8]. One study demonstrated that pancreatic cancer with isolated metastasis to the liver was associated with a worse outcome when compared to metastasis to the lung or distant lymph nodes [9]. Supraclavicular lymph node metastasis is an extremely rare occurrence, with very few cases documented in the literature. While not completely clear, the mechanism behind such metastasis may include embolization, permeation, and retrograde spread [10].

Pancreatic cancer and AIP have a lot of similar features; both are more common in the elderly, both can present as painless jaundice, present as a new diagnosis of type II diabetes mellitus, and both can cause elevated tumor markers [11]. One study showed that 2.5% of pancreatoduodenectomies performed in North America for presumed pancreatic cancer were later found to be AIP instead [12]. While pancreatic cancer usually presents as a pancreatic mass that is detected on CT scan, in rare cases, such as in our patient, CT scan can be negative, making it difficult to distinguish between the two conditions. EUS is typically more sensitive in detecting pancreatic cancer and is often used to obtain biopsies from identified lesions; however, there are reported cases where EUS failed to show the cancer, and the diagnosis was made after histopathological examination of the biopsies taken [13]. While AIP responds well to steroids, pancreatic cancer requires chemotherapy along with surgical resection in earlier stages, making it extremely important to differentiate between the two entities.

Tumor markers have limited diagnostic value in pancreatic cancer. CA 19-9 and carcinoembryonic antigen (CEA) are both associated with the disease, but they have low diagnostic sensitivity and specificity [14]. Abdominal imaging for vague abdominal symptoms is often what ultimately detects pancreatic cancer. Triphasic contrast-enhanced abdominal CT is considered the best modality for making a diagnosis, with 89%-97% sensitivity and 95% specificity [15], along with the ability to provide mapping of the surrounding vessels. With the accuracy of the CT scan, EUS with biopsy is now reserved for cases where diagnosis is uncertain, and in unresectable cases, before initiation of chemoradiation [16].

Supraclavicular lymph node metastasis from pancreatic cancer is uncommon, but when it occurs, it usually indicates advanced disease. The left supraclavicular node (Virchow’s node) drains lymph through the thoracic duct, which collects fluid from much of the abdomen. Cancer cells from the pancreas can spread through regional lymph nodes to the thoracic duct and eventually reach Virchow’s node. This type of spread reflects the body's natural drainage pathways and is most often seen with cancers of the GI tract [17,18].

Pancreatic cancer is classified into three categories: resectable, borderline resectable, and advanced. In resectable cases, the five-year post-operative survival rate is approximately 14.6%, and is higher in well-differentiated tumors and those without lymph node involvement [19]. Adjuvant chemotherapy is recommended for all patients [20]. Borderline resectable tumors are those with vessel involvement, where surgery is typically pursued only when complete resection is feasible. Treatment of advanced disease involves chemoradiation therapy along with targeted therapy.

## Conclusions

This case highlights how pancreatic cancer can present in unexpected ways-without a visible mass, mimicking AIP, and even spreading to uncommon sites like the supraclavicular lymph node. Despite unremarkable labs and subtle imaging findings, a careful diagnostic approach using EUS and biopsy helped reach the correct diagnosis. Recognizing these atypical presentations is crucial, as timely diagnosis can guide appropriate treatment and improve outcomes. Our take-home messages from this case include: Pancreatic cancer can mimic AIP, making diagnosis difficult. IgG4-related disease should be considered, but elevated IgG4 can also be seen in pancreatic cancer. EUS with core-needle biopsy is essential when imaging is inconclusive. Supraclavicular (Virchow’s) node involvement, though rare, may be the first sign of metastatic pancreatic cancer. A high index of suspicion is key in evaluating patients with vague abdominal pain and atypical imaging.
